# Study on improvement of copper sulfide acid soil properties and mechanism of metal ion fixation based on Fe-biochar composite

**DOI:** 10.1038/s41598-023-46913-3

**Published:** 2024-01-02

**Authors:** Xiao Zhang, Jinchun Xue, Huaqin Han, Yu Wang

**Affiliations:** School of Energy and Mechanical Engineering, Jiangxi University of Science and Technology, Nanchang, 330013 Jiangxi China

**Keywords:** Environmental sciences, Engineering

## Abstract

In this study, Fe modification of bamboo biochar (BC) with ferrate was used to construct a composite soil amendment based on K_2_FeO_4_-biochar (Fe-BC) system. Based on soil culture experiments, Fe-BC combined with organic–inorganic materials at the application levels of 3%, 5% and 10% to copper sulfide contaminated acid soil was studied. Adsorption kinetics experiment was used to investigate the adsorption capacity of Fe-modified biochar to heavy metal Cu. The results showed that the pH value of bamboo biochar could be increased by 1.12 units after K_2_FeO_4_ modification. Compared with the BC, the adsorption capacity of Cu^2+^ increased from 190.48 to 276.12 mg/g, which was mainly reflected in single-layer surface adsorption and chemisorption. Pore diffusion, electrostatic interaction and surface interaction are the possible mechanisms of Fe-BC interaction with Cu^2+^ ions. And the contents of Pb, Cu and Zn in soil leaching state decreased by 59.20%, 65.88% and 57.88%, respectively, at the 10% application level of Fe-BC. In general, the composite modifier based on ferrate and biochar has a positive effect on improving the characteristics of acidic soil in copper mining area.

## Introduction

Frequent mining activities have caused serious damage to the soil environment around the mine, resulting in mine-polluted soil with high acidity, high heavy metal content, and lack of nutrients^1,2^. This pollution can negatively affect soil quality and soil productivity, and even endanger animal and human health and well-being due to its impact on the food chain^3^. At present, there are many remediation methods for mine-contaminated soil, which generally include physicochemical remediation^4^, phytoremediation^5^, and bioremediation^6^. Among them, physicochemical remediation refers to the addition of different materials to improve contaminated soil in mining areas^7^. Research has shown that biochar possesses a high surface area per unit volume, a good pore structure, a high abundance of oxygen-containing functional groups, and outstanding adsorption capabilities. These properties enable biochar to raise the soil’s pH value, modify the solubility, valence, and form of heavy metals in the soil, and reduce their toxicity by immobilizing them^8–10^. In some cases, the impact of untreated biochar on enhancing soil quality is not satisfactory. In order to improve the effect of biochar remediation, scholars have studied unequal biochar modification methods, that is, through physical or chemical methods to activate the original biochar to achieve the desired purpose^11^. Common biochar modification methods include chemical oxidation^12^, chemical reduction^13^, metal impregnation^14^, etc. Saffari’s team found that coated biochar with chitosan, ball milling and humic acid has high Ni fixation potential in soil^15^. Among them, metal impregnation uses metal ions to bind to adsorbent and load them into the physical structure of biochar, comprising its surface and pore characteristics to improve its adsorption performance^11^. Metal cations commonly used for modification include iron^16^, magnesium^17^, silver^18^ and so on.

As iron is a very abundant element on Earth, developed based on nZVI^19^, iron oxides (such as ferric oxide^20^, ferric oxide^21^ and FeOOH^22,23^), iron sulfide^24^ (ferrous sulfide) and ferric chloride^25^. So far, different types of iron-modified biochar composites have been developed and targeted for soil remediation with different pollutants^26^. The nZVI@BC prepared by physicochemical method can effectively inhibit the desorption of Cd and reduce the EX and Car forms of Cd^27^. Magnetic biochar is often prepared with ferrous and iron compounds as magnetic medium precursors, but there are relatively few studies investigating the production of magnetic biochar with ferrite. Ferrate (K_2_FeO_4_) as an internal oxidation modifier, no additional modifiers or multiple operations are necessary to prepare highly adsorbable magnetic biochar using ferrite^28^. As a modifier, K_2_FeO_4_ contains the functions of oxidation activity, iron oxide loading and KOH activation^29^. Oxide-reducing groups on biochar interact with ferrates by electron shuttling, promoting the generation of intermediate iron species through electron transfer^30^. Therefore, in recent years, the combination of ferrite-biochar for pollution removal has increasingly gained attention as a research focus. For example, magnetic biochar prepared by pyrolysis of pomelo peel and ferrate can improve the adsorption performance of biochar^31^. Ferrate could significantly remove As^3+^ under the influence of straw biochar, the ferrate/biochar system achieved a removal rate of more than 91% for arsenic^32^. In addition, the treatment of sludge biochar by K_2_FeO_4_ can enhance the abundance of functional groups of sludge biochar and improve the removal effect of Pb^2+^^32^. In addition, the study found exposure to UV radiation has the potential to augment the quantity of functional groups present on the surface of biochar and improve the adsorption performance^33,34^.

Studies have found that the comprehensive improvement technology of organic–inorganic combined application is an effective method to solve the multiple problems of acid-polluted soil^35^. Organic fertilizers can effectively improve the problem of poor soil nutrients^36^. Among them, earthworm manure contains rich nutrients and a large amount of humus, and has good ventilation and water retention, which can effectively increase the level of organic matter in the soil^37,38^. Compared with lime, carbide slag exhibits a higher capacity for buffering acidity and stabilizing pollutants and potential nutrient capacity^39^. In this work, through outdoor soil culture experiment, the effects of calcium carbide slag and earthworm castings combined with Fe-biochar system on the physical and chemical properties and heavy metal content of polluted acidic soil in copper sulfide mines were studied to improve the mine soil environment conducive to plant growth, so as to achieve a relatively stable ecosystem. The immobilization mechanism of heavy metal ions in Fe-biochar system was investigated by adsorption test, which provided theoretical basis for improving copper sulfide acid soil on a large scale.

## Materials and methods

### Materials

The soil used in the experiment was taken from contaminated soil around a copper sulfide mine in Jiangxi Province of China (115° 49′ 16″ E, 29° 41′ 57″ N). Mine soil samples were collected at intervals of 10 m and at a depth of about 20 cm in the soil profile in the unvegetated mine soil zone. Before the experiment officially begins, the soil samples collected from the mining region were dried in the open air, pulverized, and treated with 100 mesh mesh. The cumulative quantity of Pb, Cu and Zn, the content of DTPA-extractable in the tested copper sulfide acid soil were shown in Table 1. All the reagents and chemicals were of analytical grade. Ultrapure water was used to formulate all the solutions for the experiments. Earthworm manure purchased from a local environmental protection technology Co. LTD. Carbide slag is derived from the waste produced in the process of acetylene production. As shown in Supplementary Fig. F1, the primary constituent of carbide slag is calcium hydroxide (Ca(OH)_2_), containing a small amount of Al and Si, and the concentration of Cu, Zn, and Pb heavy metals can be ignored compared with the content of soil in the mining area.

**Table 1:** The total amount of Pb, Cu and Zn and the content of DTPA-extractable in soil tested.

Project	ω(Pb)/(mg/kg)	ω(Cu)/(mg/kg)	ω(Zn)/(mg/kg)
Total	2333	2253	2167
DTPA-extractable content	222.88	138.45	29.66
Soil background values of Jiangxi Province	32	20.8	69
The second grade standard values (pH > 7.5)	350	100	300
The environmental quality standard for soils (pH > 7.5)	80	100	300

### Modified biochar treatment

Place the bamboo pieces in an electric blast drying oven at 90 °C for drying, then reduce them to a particle size ranging from 0.1 to 5 mm by grinding, sift them for use. The pretreated pulverized raw material was placed inside a quartz tube which was then inserted into a tube furnace filled with N_2_, and the temperature was heated to 600 °C at the rate of 10 °C/min, and the pyrolysis was performed for 5 h. And the obtained bamboo biochar has a specific surface area of 18.63 m^2^/g, an electrical conductivity of 237 mS/m, an organic carbon content of 781.2 g/kg and an ash content of 4.69%. Take 50 g bamboo biochar into 1000 mL beaker, weigh a certain mass of K_2_FeO_4_ powder and dissolve it in 500 mL deionized water, stir it with a glass rod, stir it on a magnetic mixer at 45 °C for 8 h, after stirring it, put it into an ultrasonic machine for 1.5 h, and then take out the beaker and stand. Strain and dry in an electric blast drying oven. The dried biochar was then subjected to UV irradiation using a UV lamp that emitted radiation at a wavelength of 365 nm. The biochar was placed at 60 mm from the lamp and was exposed to UV radiation for a period of 12 h. This process resulted in the formation of potassium ferrate modified biochar, which was labeled Fe-BC.

### Experimental design and treatments

The soil was placed outdoors to dry naturally, put into the crusher for crushing, and then screening was conducted using a 2 mm diameter sieve to separate and reserve only the particles that were smaller than or equal to 2 mm in size. Next put 1.5 kg of dry-weight soil into a plastic pot. Before the formal experiment, the data were consulted, and the amount of carbide slag and earthworm manure droppings was pre-tested. The application amount of carbide slag was set as 0, 1%, 2%, 3%; the dry weight of earthworm manure was 0, 3%, 5%, 7%. Preliminary experimental results show that carbide slag applied at 2% and 3%can significantly increase the pH of acidic soil, which is consistent with the conclusions of researchers^40^. However, the excessive addition of carbide slag as a solid waste will be counterproductive. The increase of organic matter content in acid soil of copper sulfide was significantly higher than that of 3% when the earthworm manure was added at 5% and 7%. Therefore, combining the economic cost and environmental cost, the application amount of carbide slag and earthworm cast is set at 2% and 5%. In this research, carbide slag and earthworm castings (dry weight) were set in two dosages, in which the dosage of carbide slag was 0 and 2% (w/w), the dosage of earthworm cast (dry weight) was 0 and 5% (w/w), and the dosage of Fe-BC was 0, 3% (w/w), 5% and 10%, and three replicates were set for each treatment, as shown in Table 2. It is then placed outdoors for soil culture. During soil cultivation, water 200 mL every 2 days and loosen the soil.

**Table 2:** The experimental scheme.

Treatment	Fe-BC (w/w)	Earthworm manure (w/w)	Carbide slag (w/w)
CK	0	0	0
F	0	5%	0
FC	3%	5%	0
FD	0	5%	2%
FDC1	3%	5%	2%
FDC2	5%	5%	2%
FDC3	10%	5%	2%

### Chemical analysis

#### Kinetic tests

##### Adsorption kinetics

The dosage of biochar and Fe-BC were both 2.0 g/L, the concentration of mass Cu^2+^ was set to 20 mg/L, using a certain concentration of HNO_3_ and NaOH to adjust the pH of the solution to about 5.5, the oscillation speed was 150 r/min, and the operating temperature was 25 °C. The samples were taken at 0, 5, 10, 20, 30, 60, 90, 120, 180, 240, 300, 360 and 480 min successively. After the adsorption experiment was completed, the conical bottle was removed, left for 30 min, and then filtered with a 0.45 μm filter membrane. The filtrate was put into the sample bottle to determine the concentration of heavy metal ions in the solution. The adsorption kinetics were fitted using the experimental results of reaction time.

##### Isothermal kinetics

The dosage of biochar and Fe-BC were both 2.0 g/L, the level of heavy metal ions was set as 10, 20, 50, 100, 200, 500, 800 and 1000 mg/L, and use a certain concentration of HNO_3_ and NaOH to adjust the pH of the solution to about 5.5. The oscillation speed is 150 r/min, the operating temperature is 298 K, and the oscillation is 300 min. After the adsorption experiment was completed, the conical bottle was removed, left for 30 min, and then filtered with a 0.45 μm filter membrane. The filtrate was put into the sample bottle to determine the heavy metal ion concentration present in the solution.

### Determination items and methods

The pH values of the tested soil and biochar were determined by pH meter (soil solid–liquid ratio 1:2.5, biochar/modified biochar solid–liquid ratio 1:30). The cation exchange capacity (CEC) of soil was measured by sodium acetate flame spectrophotometry, and the total amount of heavy metals in soil was measured by inductively coupled plasma emission spectrometry. The concentration of active heavy metals was determined by DTPA-extractable and inductively coupled plasma emission spectrometry.

### Data analysis method

The adsorption effect of biochar and Fe-BC on heavy metal ions is expressed by adsorption amount *Q*_*e*_ and removal rate *E*, and the calculation formulas are as follows,

1 $${Q}_{e}=(\left({c}_{0}-{c}_{e}\right)V)/m,$$

2 $$E=\left(\frac{\left({c}_{0}-{c}_{e}\right)}{{c}_{0}}\right)100\mathrm{\%}.$$

In the formula, when the adsorption reaches equilibrium, the adsorption capacity and heavy metal concentration are expressed by *Q*_*e*_ (mg/g) and *C*_*e*_ (mg/L), respectively.

The equation of the first-order adsorption kinetics model is,

3 $$\mathrm{ln}\left({Q}_{e}-{Q}_{t}\right)=\mathrm{ln}{Q}_{e}-{K}_{1}t,$$

the equation of the 2nd-order adsorption kinetics model is,

4 $$\frac{t}{{Q}_{t}}=\frac{1}{{K}_{2}{Q}_{e}^{2}}+\frac{t}{{Q}_{e}},$$

the Weber–Morris intraparticle diffusion model is:

5 $${Q}_{t}={K}_{p,i}{t}^{0.5}+C,$$

the Elovich model is:

6 $${Q}_{t}=\frac{1}{\beta }{\text{ln}}\alpha \beta +\frac{1}{\beta }{\text{ln}}t,$$

where *Q*_*t*_ (mg/g) and *K* (*K*_1_ is the rate constant of the 1st-order adsorption equation, min^−1^, and the rate constant for adsorption *K*_2_ is g/(mg min)) represent the adsorption amount at time t (time) and the adsorption rate constant, respectively. *K*_*p,i*_ represents the rate constant for the equation governing the diffusion of particles, mg/(g min^0.5^); *C*, *α* and *β* represent the thickness constant and Elovich constants of the adsorbent boundary layer, respectively.

The equation of the Langmuir isothermal adsorption model is:

7 $${Q}_{e}=({Q}_{m}{K}_{L}{C}_{e})/(1+{K}_{L}{C}_{e}).$$

The separation factor *R*_*L*_ defined by the Langmuir equation can be used to evaluate the degree of difficulty of adsorption:

8 $${R}_{L}=1/(1+{K}_{L}{C}_{0}).$$

When* R*_*L*_ = 0, it is irreversible adsorption. When 0 < *R*_*L*_ < 1, adsorption is straightforward. *R*_*L*_ = 1 is adsorption that can be reversed back. *R*_*L*_ > 1 indicates that adsorption is difficult^41^.

The equation of the Freundlich isothermal adsorption model is:

9 $${Q}_{e}={K}_{F}{C}_{e}^\frac{1}{n},$$

where, *Q*_*m*_ is the saturation adsorbing power, mg/g; *K*_*L*_ denotes the equilibrium constant of adsorption in the Langmuir adsorption model. *K*_*F*_ and n represent the empirical parameters of adsorption capacity and adsorption strength in model Freundlich, respectively.

SPSS19.0 software used for conducting an Analysis of Variance (ANOVA) and Duncan multiple comparisons. Plot with Origin 2021 and perform principal component analysis and redundancy analysis with Canoco 5.0 software.

## Results and discussion

### The physicochemical properties value of soil under different treatments changed dynamically

The effect of the modified material on soil improvement was evaluated by measuring and analyzing the effect on pH value, organic matter, and cation exchange capacity of copper sulfide acid soil on research.

**Figure 1:**
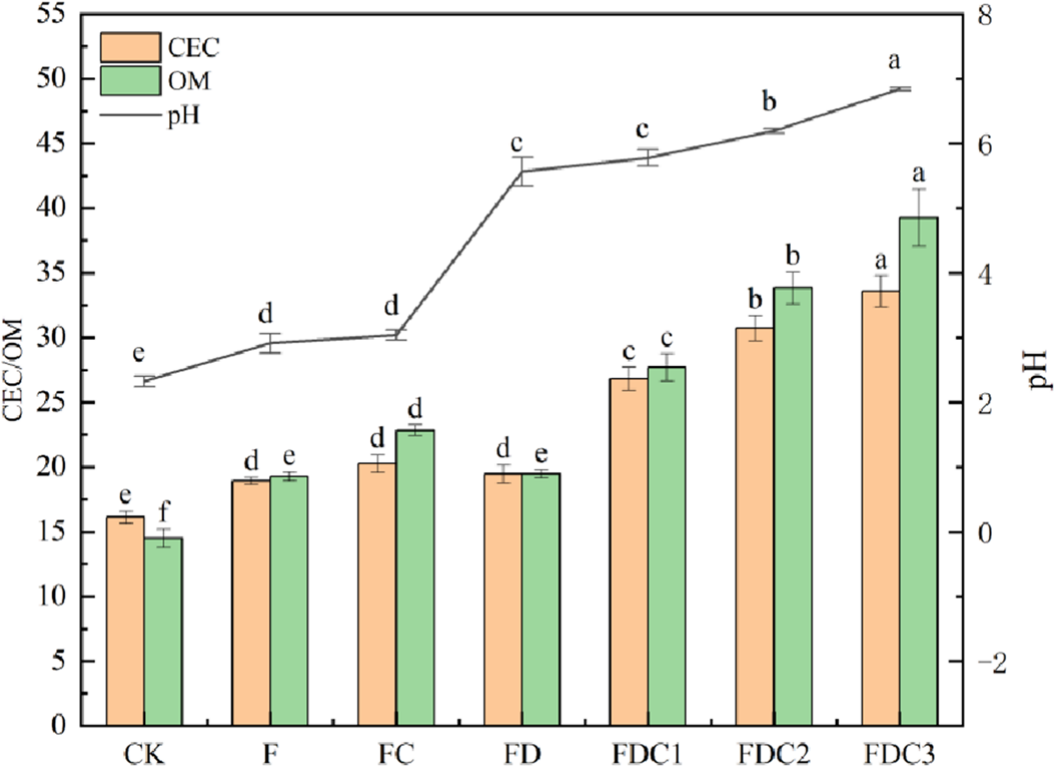
Improvement effect of the modifier on pH, cation exchange capacity and organic matter of copper sulfide acid soil.

As demonstrated in Fig. 1, as opposed to CK, the level of soil acidity in the treatment group added with improved materials significantly increased. In the intervention group, FDC3 had the most notable outcome on the increase of pH of acidic soil (*P* < 0.05), reaching 6.84. In treatment group F, adding 5% earthworm manure to soil could markedly raise the pH level of acidic soil, FC in the treatment group could substantially elevate the acidity level of soil (*P* < 0.05), but there was no substantial variation between FC and F in the exposed sample (*P* > 0.05), indicating that the application of 3%Fe-BC based on a single application of earthworm manure did not have a notable impact on the increase in pH of acidic soil. FD in the treatment group had a very significant difference in increasing the pH measurement of acidic soil samples (*P* < 0.001), and compared with CK, the increase was 3.34 units. The use of carbide slag resulted in the most significant rise in the pH of acidic soil. Research has demonstrated that the utilization of carbide slag can raise the pH level of acidic soil, possibly because calcium silicate (C–S–H), calcium hydroxide and a small amount of ettringite will be hydrated by carbide slag, increasing pH of acidic soil^42^. There was no significant difference between FDC1 (5.78) > FD (5.57) in the treatment group (*P* > 0.05). With the increase of Fe-BC application amount, the pH levels of acidic soil exhibited a noticeable upward trend, and the disparity was considerable (*P* < 0.05).

The main determinant of the soil buffer capacity is the ion exchange capacity (CEC), which holds immense importance in rational fertilization and soil enhancement. It is evident from Fig. 1 that different amendments and organic fertilizer treatments significantly affect CEC in acidic soil. Compared with CK, the addition of amendments can significantly increase CEC in acidic soil of copper sulfide by 17.49–108.31%, in which FC (20.26) > FD (19.47) > F (18.94) in treatment group. However, there were no notable discrepancies observed between the three groups subjected to different treatments (*P* > 0.05), indicating that the application of carbide slag and 3% (w/w) Fe-BC had no significant impact on the enhancement of CEC in acidic soil containing copper sulfides. With the increase of Fe-BC dosage, CEC increased by 66.38–108.31%. The CEC of acid soil in the 10% (w/w) Fe-BC treatment group increased by FDC3 to 33.58 cmol/kg.

Copper mining area contaminated low pH soil is poor in texture and lacks nutrients. The study discovered that the organic matter concentration in each treatment group was significantly increased by adding soil improvement materials. The results showed that the mass fraction of organic matter in acid soil increased by 32.83–170.55% after the application of improved materials. Among them, under the implementation of 5% (w/w) earthworm manure and 2% (w/w) carbide slag, 10% (w/w) Fe-BC increased the organic matter content of acid soil the most, reaching 39.25 g/kg, which was 2.71 times higher than CK, and the improvement effect was considerable. The application of organic fertilizer earthworm manure could significantly increase the content of organic matter in acid soil (*P* < 0.05). As a kind of alkaline solid waste, carbide slag had no significant difference in organic matter content between F and FD among treatment groups (*P* > 0.05), indicating that carbide slag had no obvious effect on the improvement of organic matter in acidic soil. In addition, with the increase of Fe-BC application amount, soil organic matter content showed an increasing trend, and there were notable variations between the groups that received different treatments (*P* < 0.05).

### Effects of different treatment groups on heavy metals in soil

#### Effects of different treatment groups on total heavy metal content in soil

The content of heavy metals in acidic soil of copper sulfide mine far exceeds the prescribed limit of heavy metals in the soil of the specific region (as shown in Table 3). The contents of Pb, Cu and Zn in soil were 2.33 g/kg, 2.25 g/kg and 2.17 g/kg, respectively. The total amount of Pb, Cu and Zn decreased by 3.43–11.21%, 2.22–19.56% and 2.77–11.98%, respectively. The application of earthworm manure alone increased the content of heavy metal Zn in soil slightly, but there was no notable discrepancy detected (*P* > 0.05). The combination of earthworm cast and carbide slag with earthworm castings and 3% (w/w) Fe-BC combined application could reduce the overall heavy metal content in soil, but the diversity between the two groups was not statistically significant. (*P* > 0.05). The concentration of Pb, Cu and Zn in soil of mine was decreased, and 10% (w/w) Fe-BC addition was the most evident way to reduce the total Pb, Cu and Zn content in soil.

**Table 3:** The improvement impact of soil amendments on the total amount of heavy metals in copper sulfide acid soil.

	CK	F	FC	FD	FDC1	FDC2	FDC3
Pb	2.33 ± 0.072a	2.21 ± 0.062bc	2.18 ± 0.017bc	2.25 ± 0.060ab	2.11 ± 0.025cd	2.07 ± 0.032de	1.98 ± 0.042e
Cu	2.25 ± 0.045a	2.20 ± 0.021a	2.10 ± 0.012b	2.11 ± 0.038b	2.10 ± 0.025b	1.97 ± 0.040bc	1.81 ± 0.031d
Zn	2.17 ± 0.032ab	2.18 ± 0.036a	2.08 ± 0.046c	2.11 ± 0.055bc	2.09 ± 0.025c	1.98 ± 0.029d	1.91 ± 0.021d

The data are presented as mean ± standard deviation. Different letters in the same column signify significant variations among the treatments (*P* < 0.05).

#### Changes of DTPA-extractable contents of Pb, Zn and Cu after treatment with different materials

##### Change of DTPA-extractable Pb content

**Figure 2:**
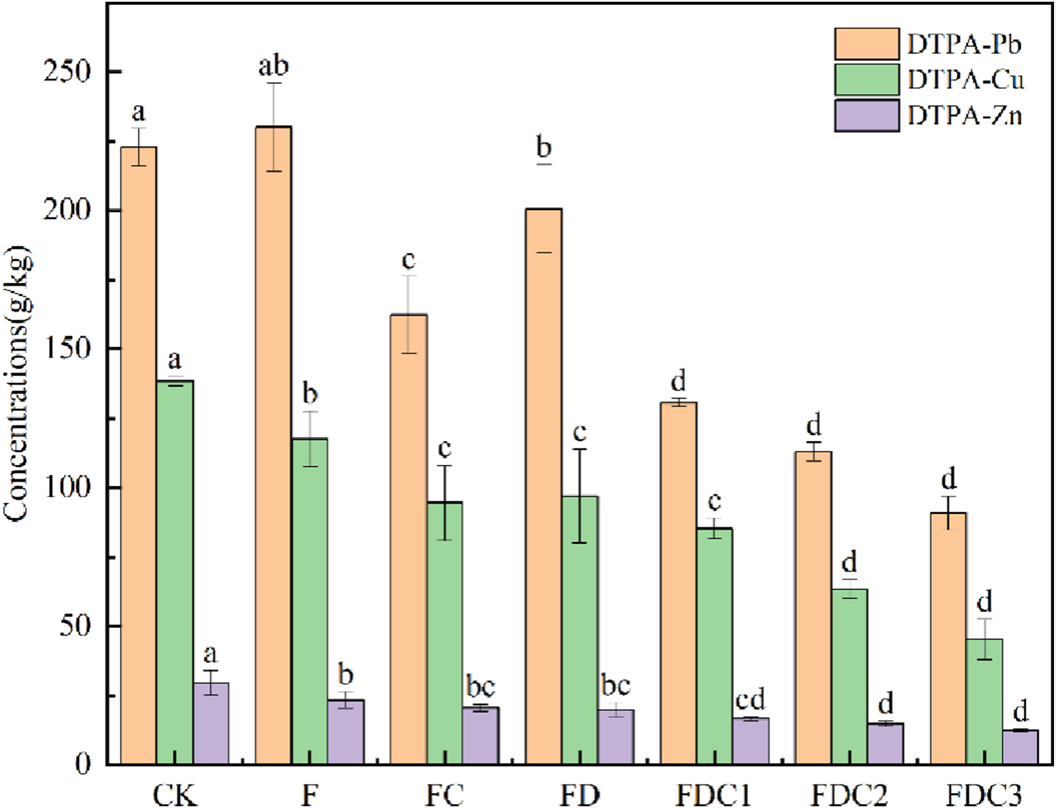
Changes of available content of heavy metals in soil of different treatment groups.

After adding different repair materials, the change of Pb content extractable by DTPA in acid soil is revealed in Fig. 2. From the perspective of the 60-day repair effect, after using different repair materials, the leachable Pb content in acidic soil decreased to different degrees, but the decrease was different: FDC3 reduced the leachable Pb content in acidic soil by 59.20%, which was relatively large, and the repair effect was obvious. Followed by FDC2, FDC1, decreased by 53.76%, 49.89%.

##### Change of DTPA-extractable Zn content

After adding different repair materials, the changes in content of Zn extracted by DTPA in acidic soil are illustrated in Fig. 2. According to the repair effect of 60 days, after using different repair materials, the concentration of leach Zn in acidic soil decreased to different degrees, but the decrease was different: FDC3 reduced the content of leachable Zn content in acidic soil by 57.88%, which was relatively large, and the repair effect was obvious. It was followed by FDC2 and FDC1, which decreased by 50.10% and 43.70% respectively.

##### Change of DTPA-extractable Cu content

After adding different repair materials, the change of DTPA-extracted Cu content in acidic soil is shown in Fig. 2. According to the repair effect of 60 days, after using different repair materials, the leaching Cu content in acidic soil decreased to different degrees, but the decrease was different: FDC3 reduced the leachable Cu content in acidic soil by 65.88%, which was relatively large, and the repair effect was obvious. Followed by FDC2, FDC1, decreased by 51.77%, 33.51%, respectively.

#### Principal component analysis (PCA) and redundancy analysis (RDA) of heavy metals in soil

**Figure 3:**
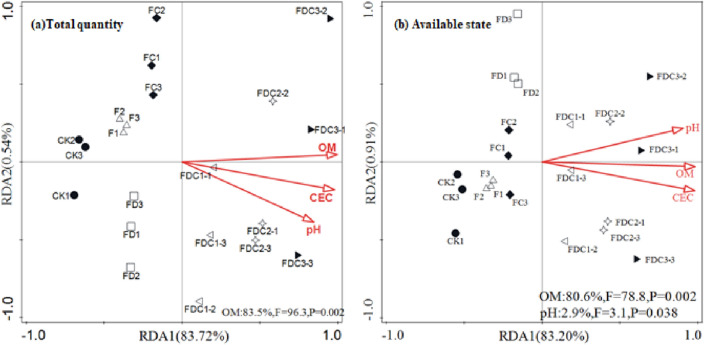
RDA of the correlations between soil factors with total and available soil heavy metals.

PCA was carried out for the total amount and the available states of the three heavy metals, the first two principal components of the full amount and available state of heavy metals accounted for 91.68% and 89.10% of the total variance Supplementary Fig. F2. The addition of soil amendments markedly changed the total amount and available state of Pb, Cu and Zn in acidic soil, as can be seen from the distribution diagram of principal components. For the overall quantity of Pb, Cu and Zn in soil, blank group CK is located in the third quadrant, single addition of earthworm manure (F), earthworm manure and carbide slag (FD), earthworm manure and Fe-BC (FC) treatment is distributed in the second and third quadrant, while earthworm manure, carbide slag and Fe-BC combined treatment is distributed in the first and fourth quadrant, and with the increase of Fe-BC dosage, CK treatment is distributed in the third quadrant. The distribution of sample points moves from the upper left of the first quadrant to the lower right of the fourth quadrant. Through RDA, it was found that there was a strong constraint relationship between the total amount and available state of Pb, Cu and Zn in soil and soil parameters. 84.26% of the overall variability of Pb, Cu and Zn in soil could be explained by soil pH, CEC, and OM, among which OM explained 83.5% of the variance variation, reaching a significant level (Fig. 3a). 84.11% of the variation in the available state of Pb, Cu and Zn in soil can be attributed to soil pH, CEC, and OM, in which soil OM and pH explain 80.6% and 2.9% of the variance variation, respectively, reaching a significant level (Fig. 3b). The results of RDA showed that the total amount and available state of Pb, Cu and Zn in soil was strongly influenced by soil pH and organic matter.

### Adsorption experiment of Cu^2+^ by iron-modified biochar

Adsorption technology plays a good role in removing heavy metals, the adsorption experiment is convenient to operate and is conducive to the recovery of metal pollutants. With the objective of researching the adsorption kinetics of BC and Fe-BC on heavy metal ions, this research took the adsorption of Cu^2+^ as an example and determined the corresponding adsorption kinetics model. In this experiment, pseudo-1st order kinetics, quasi-2nd order kinetics, Langmuir adsorption equation and Freundlich isothermal adsorption equation, Weber–Morris intraparticle diffusion model and Elovich model were utilized for curve-fitting the kinetic data, to explain adsorption mechanism and determine the reaction order and elucidate the adsorption mechanism of the adsorption process.

#### Adsorption kinetics experiment

The impacts of BC and Fe-BC adsorption on Cu^2+^ at different contact times within 480 min are shown in Supplementary Fig. F3. As shown in the figure, the adsorption of Cu^2+^ by Fe-BC mainly went through three stages: accelerated, slow and balanced, and the equilibrium of adsorption was reached after 300 min of adsorption. Within 30 min of the reaction, the adsorption efficiency of biochar is fast, possibly because abundant minerals and surface electron-contributing groups in the material provide a large number of adsorption sites to bind to Cu^2+^^43^. Nonetheless, as the duration of adsorption increased, the available adsorption sites on the surface of biochar became saturated, and the increased rate of adsorption decreased gradually. The adsorption of Fe-BC to Cu^2+^ reached equilibrium at about 300 min, and the adsorption of Cu^2+^ by BC reached equilibrium at about 240 min. In the case of Fe-BC and BC, the *Q*_*e*_ of Fe-BC for Cu is 25.92 mg/g, which is higher than that of BC for Cu at 16.38 mg/g. To further investigate the adsorption mechanism, pseudo-1st order kinetics and quasi-2nd order kinetics were applied for model the adsorption process of Fe-BC. The fitting-related parameter are depicted in Table 4. As depicted in the table, the correlation coefficients obtained from fitting (*R*^2^) of the quasi-2nd order kinetic model for the adsorption of Cu^2+^ by BC and Fe-BC are both greater than the pseudo-1st order kinetic model, and the *R*^2^ values of the quasi-2nd order kinetic model are both superior to 0.98. Therefore, the adsorption processes of the heavy metal Cu^2+^ by BC and Fe-BC are more congruent with the quasi-second-order kinetic model. The data demonstrates that the uptake of Cu^2+^ by Fe-BC is double nuclear adsorption, and there are various adsorption nodes on the exterior of Fe-BC, which can be chemically adsorbed with heavy metal ions by ionic or covalent bonds, indicating that there may be ion exchange, complexation and precipitation between Fe-BC and Cu^2+^, and the adsorption process of Fe-Bc on Cu^2+^ is controlled by chemisorption.

**Table 4:** Parameters of adsorption kinetics model of BC and Fe-BC for Cu^2+^

Adsorption material	Heavy metal	${Q}_{e}$(mg/g^−1^)	First-order rate constants			Second-order rate constants		
			${Q}_{e}$/(mg/g)	${K}_{1}$	${R}^{2}$	${Q}_{e}$/(mg/g)	${K}_{2}$	${R}^{2}$
Fe-BC	Cu^2+^	25.92	20.32	0.0120	0.9406	30.30	0.0006	0.9928
BC	Cu^2+^	16.38	12.04	0.0013	0.9667	18.02	0.0015	0.9843

#### Adsorption isotherm experiment

The fitted model parameters and adsorption isotherms were shown in Supplementary Fig. F3 and Supplementary Table S1. It is clear from the table, the Langmuir model’s correlation coefficient (*R*^2^ = 0.9936) significantly outperforms that of the Freundlich model (*R*^2^ = 0.9370, *K*_*L*_ = 20.746), demonstrating its more accurate match with the experimental results. That is, the adsorption process of Fe-BC adsorbing Cu^2+^ belongs to single molecular layer adsorption, so one adsorption point can only accommodate one adsorbed ion, and all adsorption sites have the same adsorption performance. The *Q*_*m*_ = 276.12 mg/g is higher than the maximum adsorption capacity of BC adsorbing Cu^2+^ (*Q*_*m*_ = 190.48 mg/g). This may improve the adsorption performance of the composite material after iron modification with biochar so that the adsorbent has a better adsorption effect on heavy metal. In addition, it is calculated that the separation factors *R*_*L*_ corresponding to the Langmuir equation in this study are all between 0 and 1, indicating that the adsorption reactions of BC, Fe-BC and Cu^2+^ are easy to carry out.

#### Weber–Morris intraparticle diffusion model

Because of the distributed adsorption characteristics of iron-modified biochar on heavy metal ions, it is inferred that the adsorption process contains multiple complex effects, so the in-particle diffusion model is used to supplement the dynamic models. Weber–Morris intraparticle diffusion model can reflect the actual velocity control steps and the corresponding reaction mechanism in the adsorption process. The fitting results for iron-modified adsorption of heavy metal Cu^2+^ are depicted in Supplementary Fig. F3 and Supplementary Table S2. The figure illustrates that the fitting line of *Q*_*t*_ to *t*^0.5^ does not pass through the origin, pointing to the fact that diffusion within the particle is not the only limiting factor for the adsorption rate^44^. In other words, the process of adsorption of Fe-BC for heavy metal is divided into two stages: particle membrane diffusion and intra-particle diffusion, additionally, the presence of reactions can alter the adsorption rate such as ion exchange and precipitation^45^. A large slope of the fitted line in stage 1 indicates the process of diffusion of heavy metal ions onto the surface of Fe-BC through a solution, while a decrease in the slope of the fitted line in stage 2 indicates the process of diffusion of adsorbent into the adsorbent through micropores at the Fe-BC surface. From stage 1 to stage 2, the diffusion resistance may be increased as Cu^2+^ diffuses into the adsorbent. The diffusion rate is reduced. Finally, the adsorption equilibrium state is reached. The Table S2 demonstrates that the parameters of the particle diffusion model of Fe-BC for Cu^2+^ are *K*_*p*,1_ > *K*_*p*,2_ and *C*_1_ < *C*_2_, the fact that heavy metal Cu^2+^ in solution is mainly adsorbed by Fe-BC in the initial stage of adsorption is indicated. This is consistent with the actual test process.

#### Elovich model

The biological linear fitting results of the Elovich adsorption equation for Fe-BC adsorption of heavy metal Cu^2+^ are shown in Supplementary Fig. F3 and Supplementary Table S3. There is a significant linear relationship between *Q*_*t*_ and *lnt*, and the fitting coefficient *R*^2^ = 0.9870, indicating that Fe-BC adsorption of Cu^2+^ has the kinetic characteristics of the Elovich adsorption equation. It is explained that the surface adsorption energy of Fe-BC is uniformly distributed during the whole adsorption process.

### Study on mechanism of adsorption of heavy metal ions by iron-modified biochar

In this study, Fe-BC adsorption Cu^2+^ is an example, through the determination of Fe-BC pH value. By measuring the Fe-BC surface particles with XPS, the chemical makeup of the Fe-BC surface particles can be determined, to better understand the adsorption mechanism and the source of excellent properties. The use of SEM can be used to analyze the surface morphology and microstructure of materials, and the analysis of elements in Fe-BC particles by energy dispersive EDS can determine the content of Fe and other impurity elements in the particles, leading to a better understanding of their physical and chemical properties. FTIR can be used to better understand the chemical composition and structure of Fe-BC particles, and further understand their properties and adsorption mechanism.

#### pH value

The pH value of BC is 9.63, and the pH value of Fe-BC is 10.75, which is strongly alkaline. Following the incorporation of the adsorbent (BC/Fe-BC), the pH of the soil environment will be changed by adsorption of H^+^, creating an optimal alkaline condition for heavy metal ions, which will induce the formation of metal hydroxide precipitates and enhance the adsorption of heavy metal ions by the adsorbent.

#### SEM–EDS spectra

Supplementary Figure F4 shows the SEM–EDS spectra before and after Fe-BC adsorption of Cu^2+^. It is evident from Supplementary Fig. F4 that the surface of Fe-BC is rough and dispersed with fine fragments and many pores, and the surface comprises numerous granular materials and pores, indicating that iron-containing materials are fixed on the biochar. After Cu^2+^ was adsorbed by Fe-BC, the pores of biochar were filled with granular matter, indicating that the biochar’s surface and pores adsorbed Cu^2+^.

The elements, distribution, and content of microregions were analyzed by EDS and SEM. Supplementary Figure F5 EDS energy spectrum and Fe-BC surface element distribution map obtained by using the Mapping function. The results clearly demonstrate of EDS spectrum analysis that Fe-BC after adsorption contains Cu, Fe, and other elements respectively, indicating that heavy metal Cu is adsorbed by Fe-BC. In addition, it can be seen from the EDS spectrum that Fe-BC contains a large amount of K element. Research has indicated that metal ions like K^+^ and Na^+^ present in biochar can be attracted to form metal complexes (–COOM–) due to electrostatic attraction. These metal complexes can be exchanged with other heavy metal ions in the solution, thus achieving the removal of heavy metal ions^46^. One can observe from Supplementary Fig. F5 that Fe-BC contains many C and O elements, among which the proportion of O elements increases from 18.72% before modification to 39.37%, which may be due to the reaction of biochar with potassium ferrate to produce iron oxides. After modification, Fe can be detected, accounting for 8.07% of the mass of Fe and 18.99% of the mass of K. Studies have shown that the existence of K can enhance the adsorption of heavy metals to a certain extent^47^. Through the energy spectrum analysis of Fe-BC adsorbed with Cu^2+^, the presence of Cu elements can be detected on the surface of Fe-BC material, in which the proportion of C, O and Cu elements is 33.24%, 18.72% and 31.89%, respectively.

#### FTIR spectra

The infrared spectrum of the modified material Fe-BC is shown in Supplementary Fig. F6. In the infrared spectrum of Fe-BC, the stretching vibration peaks corresponding to Fe–O can be observed at 469.65 cm^−1^ and 619.65 cm^−1^, and the bending vibration of Fe–OH at 883.51 and 786.78 cm^−1^ is the identifying peak of FeOOH. The conjugation of ketone and quinone results in the formation of peaks related to aromatic hydrocarbon C=C and C=O stretching vibrations in the proximity of 1648.98 cm^−1^ and quinone can be understood as the increase of Fe-BC carboxyl functional groups by ultraviolet lamp irradiation. The peak near 1012.93 cm^−1^ is generated by the stretching vibration of aliphatic compound C–O^48^, indicating that the biochar contains uncarbonized fatty acids. The hydroxyl group shows a deformation vibration at 1384.34 cm^−1^ as indicated by the peak observed at this frequency^49^. 1444.55 cm^−1^ was generated by the stretching vibration of the aromatic compounds –COOH– and –CHO in lignin^50^. The strong hydrogen-bonded hydroxyl absorption peak near 3398.49 cm^−1^ is a characteristic peak formed by the absorption of water molecules on the surface of Fe_2_O_3_^51^. The successful loading of K_2_FeO_4_ on biochar was demonstrated by EDS spectra.

To further determine the adsorptive mechanism of Cu^2+^ on the adsorbent, the surface state of Cu^2+^ adsorbed by Fe-BC was studied by FTIR. After the absorption of Cu^2+^ by Fe-BC, the peak of absorption of the infrared spectrum does not shift significantly, but the absorption peak intensity decreases, the amplitude decreases significantly, and the wave crest becomes slightly wider after the absorption of copper ion, indicating that the original π-conjugated aromatic structure forms a stable structure with low energy with copper ion^48^.

#### XPS spectrum

**Figure 4:**
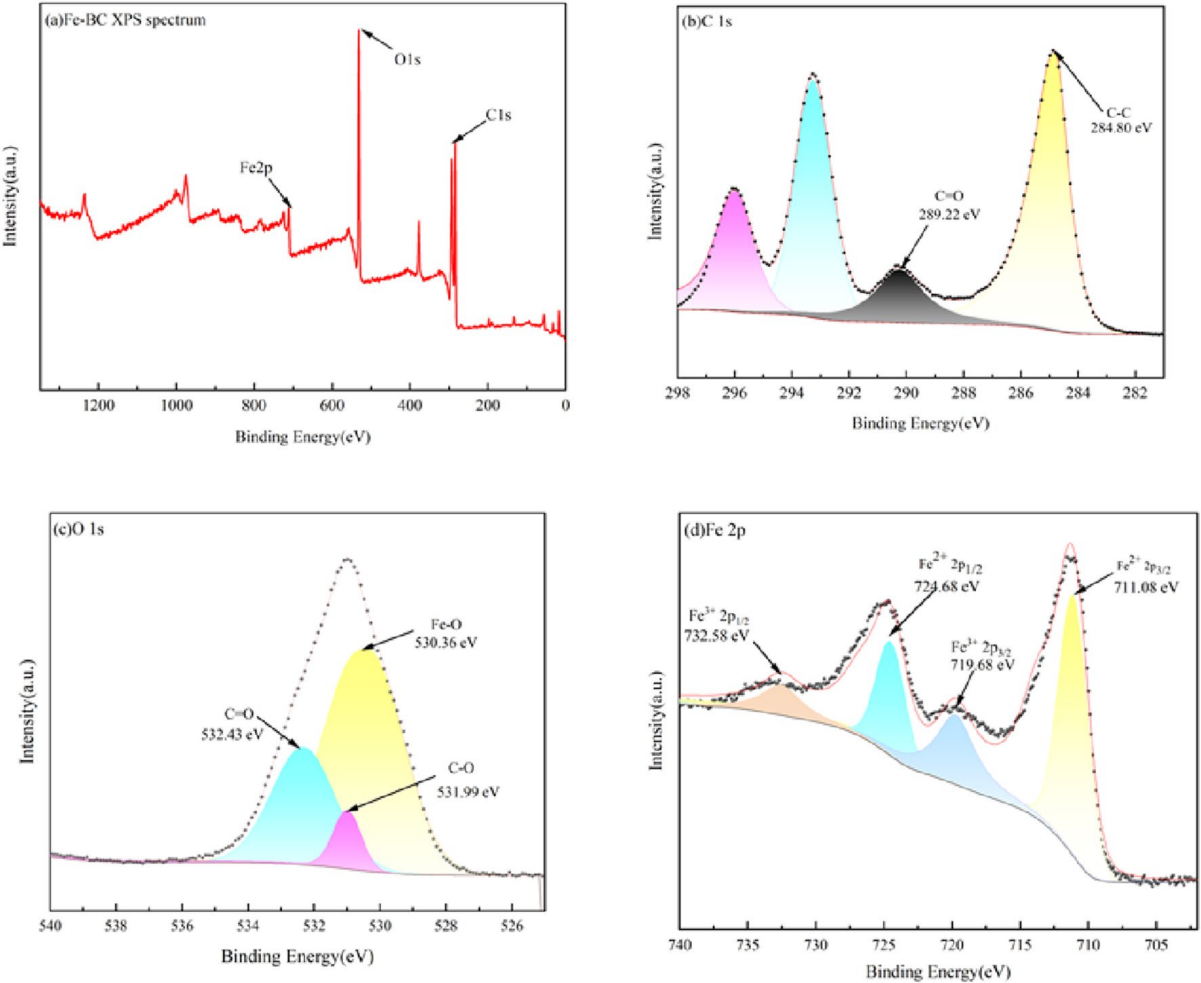
XPS full spectrum (**a**) and element fine spectrum (**b–d**) of Fe-BC.

The chemical makeup or chemical composition and compounds on the surface of iron-modified biochar composites were further analyzed by XPS. Figure 4a shows the full spectrum scan of Fe-BC, and the binding energy peaks of Fe2p appear around 710 eV. In Fig. 4d, the two peaks of Fe2p near 711.08 eV and 724.68 eV represent Fe^2+^2p_3/2_ and Fe^2+^ 2p_1/2_, respectively. The Fe^3+^2p_3/2_ and Fe^3+^2p_1/2_ are respectively depicted by the two peaks around 719.68 eV and 732.58 eV, indicating the presence of Fe_2_O_3_, Fe_3_O_4_^52^, and FeOOH in Fe-BC. The sub-peak at 706.7 eV corresponds to the binding energy of Fe^0^^53^, and the sub-peak results show that the content of Fe^0^ in the material is nearly zero, indicating that iron mainly exists in the form of Fe(II) and Fe(III). As shown in Fig. 4c, the highest peak of O1s appears near 530 eV, corresponding to metal oxide (O^2−^), and the appearance of O^2−^ peak indicates the presence of Fe_2_O_3_ in the prepared composite Fe-BC^54^. Biochar reacts with ferrate, promoting the degradation of ferrate, and increasing the production of other valence iron during the reaction. Studies have shown that ferrates form Fe_2_O_3_ and FeOOH particles in the reaction of oxidizing organic pollutants and heavy metal ions^55^. As shown in Fig. 4b, C–C function keys appear near 284.8 eV, and C=O functional groups appear near 28.9.22 eV. In addition, there are miscellaneous peaks about C. When exposed to water, ferrates will become unstable and decompose into Fe^5+^ and Fe^3+^. The Fe_2_O_3_ produced by ferrate reduction is different from the Fe_2_O_3_ produced in the biochar-ferrate. The Fe_2_O_3_ produced by ferrate reduction is different from the iron oxide produced in the biochar ferrate system, and in this combined system, reducing functional groups (phenol hydroxyl group) rich in biochar can be oxidized into oxidizing functional groups (carboxyl group), and the aromatic hydrocarbon rich portion of the biochar can provide electrons to ferrates in this system. Biochar not only enhances the generation of different valence iron valence state during the biochar/ferrate reaction, but also impacts the characteristics of the resulting iron oxides^56^.

The pore diffusion effect is mainly physical adsorption. According to SEM, the specific surface area and developed porosity texture of Fe-BC can reduce the steric hindrance effect and increase the number of sites available for Cu^2+^ adsorption, thus having a higher physical adsorption capacity^57^. The adsorption kinetics of biochar for Cu^2+^ and the rapid adsorption in the early stage of adsorption isotherm is also attributed to pore diffusion. Therefore, pore diffusion is also one of the important mechanisms of Fe-BC removal of Cu^2+^. It is apparent from the FTIR spectra that during Cu^2+^ sorption by Fe-BC, many hydroxides are consumed, leading to a significant decrease in the –O–H signal near 1384 cm^−1^, and the strong complexation between Cu^2+^ and iron oxides leads to a significant change in the peak at 1384 cm^−1^. Secondly, Fe-BC contains a variety of mineral ions, such as OH^−^, CO_3_^2−^, and SO_4_^2−^ plasma can form precipitation between Cu^2+^, and it is found by XRD analysis that Fe-BC forms Cu_4_(SO_4_)(OH)_6_⋅2H_2_O on the surface after adsorption of Cu^2+^. Fe-BC is rich in –COOH– and there is a complex effect between Cu^2+^ and Fe-BC. The negative charge of Fe-BC can easily form electrostatic interaction with Cu^2+^. The aromatic structure of Fe-BC can have a cation-π mechanism with Cu^2+^. In addition, biochar is rich in reducing functional groups after Fe modification, and Cu^2+^ is reduced to Cu^1+^ or Cu^0^. In fact, biochar materials have more adsorption mechanisms for heavy metal ions (see Supplementary Fig. F7).

## Conclusion

In this study, a new method was used to prepare iron-modified biochar, and a new composite material under Fe-biochar system was proposed. Through the experiment of Fe-biochar system combined with organic fertilizer earthworm manure and alkaline industrial waste calcium carbide slag to improve the acid soil polluted by copper sulfide. The research shows that the composite material under the Fe-biochar system can significantly increase the pH value of acidic soil and improve the soil CEC and organic matter environment. At the same time, it can effectively fix metal ions (Cu, Pb, Zn) in the soil. Combined with the Weber–Morris diffusion model and Elovich model, it is revealed that there is a multi-level mechanism of ferrite-biocarbon on the improvement of acidic mineral soil. PCA and RDA analysis showed that the total amount and availability of heavy metals in acidic soil were positively correlated with soil pH and organic matter. In summary, the new soil amendment of composite materials under Fe-biochar system can improve the quality of mine soil polluted by copper sulfide and low pH level. In addition, ferricate-biochar can be considered as an effective heavy metal fixation adsorbent. These findings help develop sustainable and effective strategies to mitigate the environmental impacts of copper sulfide mining activities.

### Supplementary Information

Supplementary Information.

## Data Availability

The authors confirm that the data supporting the findings of this study are available within the article. The authors will supply the relevant data in response to reasonable requests. For further data on this study, please contact Xiao Zhang (zxiao0801@163.com).
