# Supplementary material for: Study on improvement of copper sulfide acid soil properties and mechanism of metal ion fixation based on Fe-biochar composite
Source: Sci Rep. 2024 Jan 2;14:247. doi: 10.1038/s41598-023-46913-3 (PMC10762084; doi:10.1038/s41598-023-46913-3)
Supplement: Supplementary file 1 — Supplementary Information. [file 41598_2023_46913_MOESM1_ESM.docx]

Supplementary Figure F1：


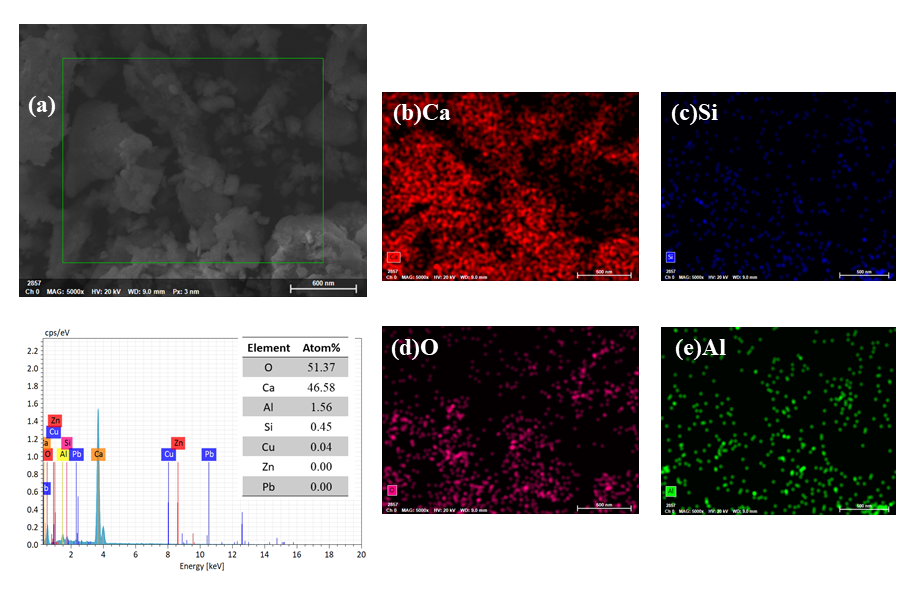


Fig. F1 SEM and EDS analysis diagram of carbide slag

Supplementary Figure F2：


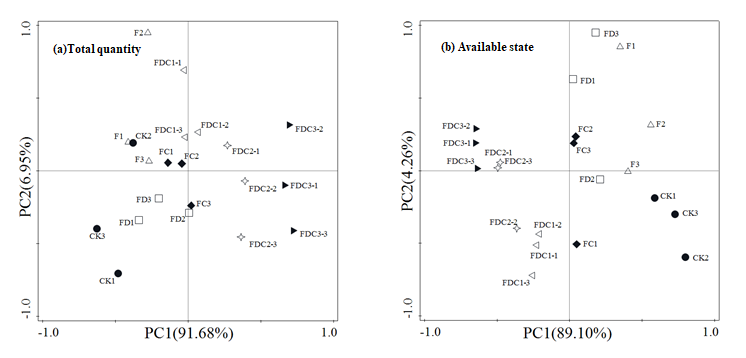


Fig. F2 PCA of total heavy metals and available heavy metals in soil under different treatments

Supplementary Figure F3：


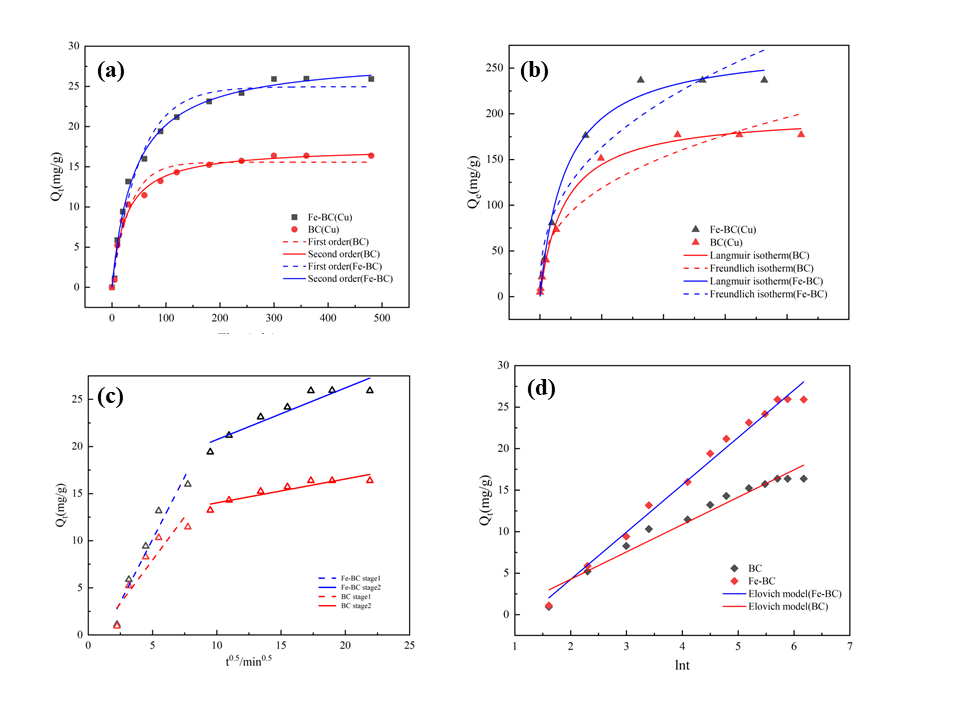


Fig. F3 Adsorption model of Cu^2+^ by BC and Fe-BC

Supplementary Table S1：

Table S1 Langmuir and Freundlich model parameters of adsorption of Cu^2+^ by BC and Fe-BC

| Adsorption material | Heavy Metal | Langmuir isotherm | | | Freundlich isotherm | | |
| --- | --- | --- | --- | --- | --- | --- | --- |
|  |  | $Q_{m}$(mg/g) | $K_{L}$ | $R^{2}$ | $K_{F}$ | $1/n$ | $R^{2}$ |
| Fe-BC | Cu^2+^ | 276.12 | 0.00120 | 0.9936 | 20.746 | 0.3894 | 0.9370 |
| BC | Cu^2+^ | 190.48 | 0.01904 | 0.9940 | 7.871 | 0.5038 | 0.9372 |

Supplementary Table S2：

Table S2 Data parameters of in-particle diffusion kinetics of adsorption of Cu^2+^ by BC and Fe-BC

| Adsorption material | Heavy Metal | Stage 1 | | | | Stage 2 | | |
| --- | --- | --- | --- | --- | --- | --- | --- | --- |
|  |  | $C_{1}$ | $K_{p,1}$/[mg/(g·min^0.5^)] | $R^{2}$ | $C_{2}$ | | $K_{p,2}$/[mg/(g·min^0.5^)] | $R^{2}$ |
| Fe-BC | Cu^2+^ | 3.1583 | 2.6571 | 0.9357 | 15.2360 | | 0.5489 | 0.8561 |
| BC | Cu^2+^ | 1.2379 | 1.8360 | 0.8070 | 11.4824 | | 0.2535 | 0.8176 |

Supplementary Table S3**：**

Table S3 Data parameters of the Elovich model of BC and Fe-BC on Cu^2+^

| Adsorption material | Heavy Metal | Elovich model | | |
| --- | --- | --- | --- | --- |
|  |  | α | β | $R^{2}$ |
| Fe-BC | Cu^2+^ | 1.6237 | 0.1754 | 0.9870 |
| BC | Cu^2+^ | 1.6292 | 0.3037 | 0.9537 |

Supplementary Figure F4：


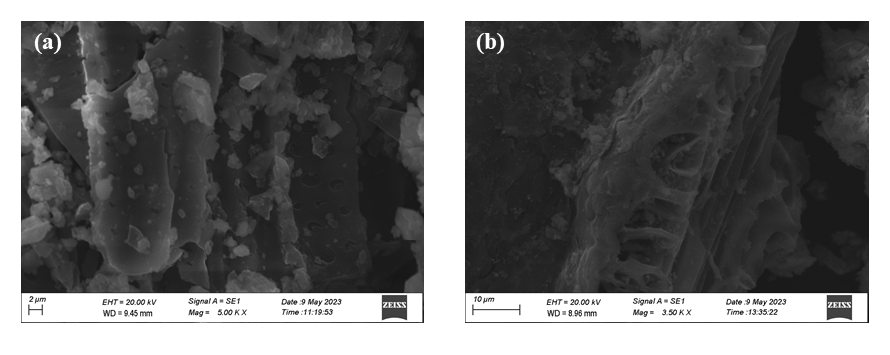


Fig. F4 SEM of Fe-BC(a) and Fe-BC adsorbing Cu(b)

Supplementary Figure F5：


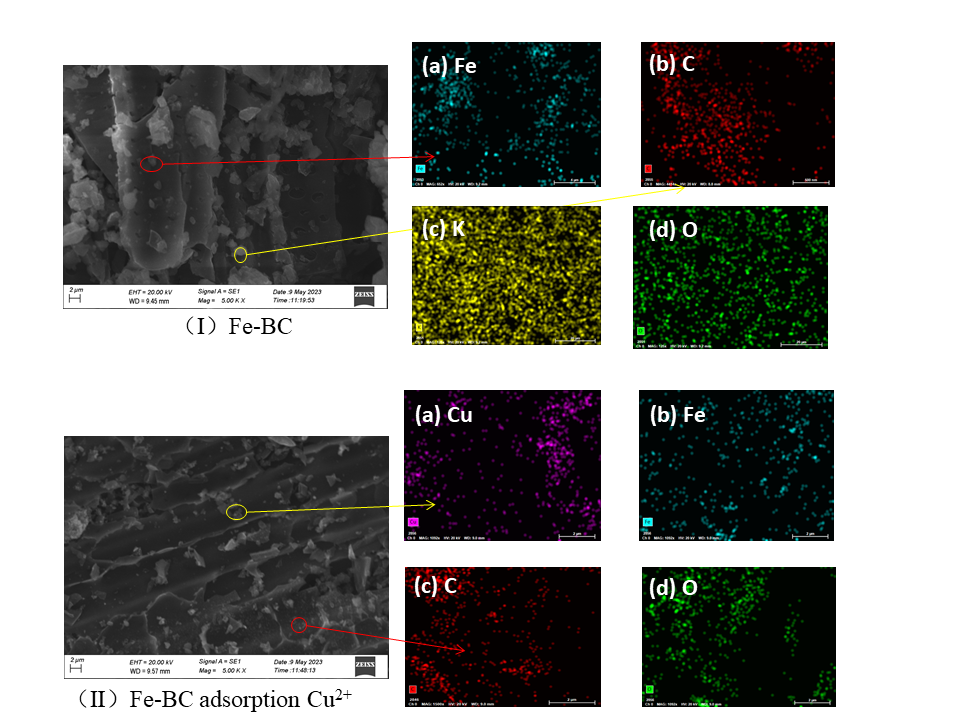


Fig. F5 Scanning electron microscopy and energy spectrum analysis before and after Fe-BC adsorption of Cu^2+^

Supplementary Figure F6：


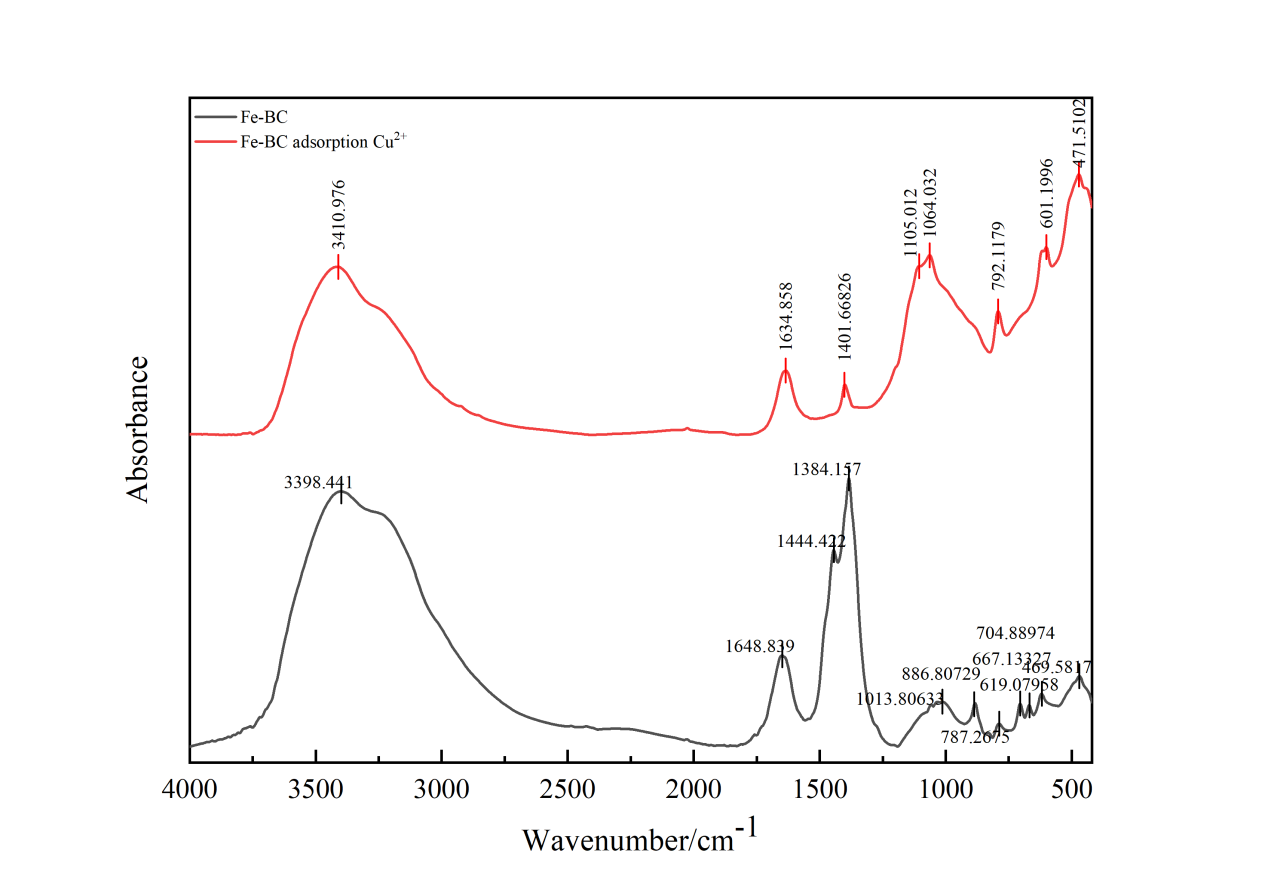


Fig. F6 FTIR spectra of Fe-BC before and after adsorption of Cu^2+^

Supplementary Figure F7:


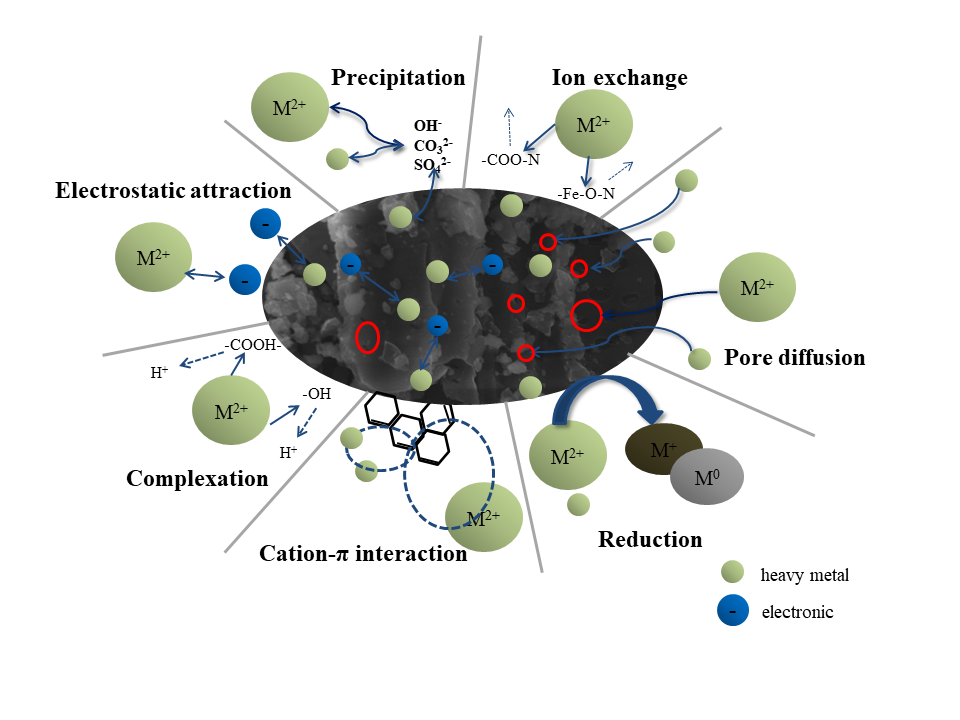


Fig. F7. Adsorption mechanism of Fe-BC on Cu^2+^
